# Significance of Wnt/β-Catenin Signal Activation for Resistance to Neoadjuvant Chemoradiotherapy in Rectal Cancer

**DOI:** 10.3390/biomedicines11010174

**Published:** 2023-01-10

**Authors:** Shoji Miyako, Takeru Matsuda, Yu-ichiro Koma, Takahiro Koide, Ryuichiro Sawada, Hiroshi Hasegawa, Kimihiro Yamashita, Hitoshi Harada, Naoki Urakawa, Hironobu Goto, Shingo Kanaji, Taro Oshikiri, Yoshihiro Kakeji

**Affiliations:** 1 Division of Gastrointestinal Surgery, Department of Surgery, Kobe University Graduate School of Medicine, Kobe 650-0017, Japan; 2 Division of Minimally Invasive Surgery, Department of Surgery, Kobe University Graduate School of Medicine, 7-5-2 Kusunoki-chou, Chuo-ku, Kobe 650-0017, Japan; 3 Division of Pathology, Department of Pathology, Kobe University Graduate School of Medicine, Kobe 650-0017, Japan; 4 Department of Surgery, Sanda City Hospital, Sanda 669-1321, Japan

**Keywords:** rectal cancer, β-catenin, NACRT

## Abstract

Although a therapeutic response to neoadjuvant chemoradiotherapy (NACRT) is important to improve oncological outcomes after surgery in patients with locally advanced rectal cancer, there is no reliable predictor for this. The Wnt/β-catenin signal is known to be crucial for the tumorigenesis of colorectal cancer. This study aimed to investigate the association of Wnt/β-catenin signal activation with a pathological response to NACRT. The immunohistochemical expression of nuclear and membranous β-catenin was analyzed in biopsy samples obtained from 60 patients with locally advanced rectal cancer who received curative surgery following NACRT. The association of Wnt/β-catenin signal activation with their clinical outcomes was investigated. Notably, the body mass index of these patients was significantly higher in the low nuclear β-catenin expression group. Moreover, patients in the high nuclear β-catenin expression group tended to have more advanced disease and a higher rate of positive vascular invasion than those in the low expression group. Furthermore, the rate of good histological responses was significantly higher in the low nuclear β-catenin expression group (72% vs. 37.1%, *p* < 0.01). Overall, relapse-free survival tended to be better in patients with low nuclear/high membranous β-catenin expression (*n* = 9) than in other individuals (*n* = 51) (*p* = 0.093 and *p* = 0.214, respectively). Activation of the Wnt/β-catenin signal pathway represented by nuclear β-catenin accumulation was significantly associated with a poor response to NACRT in patients with rectal cancer. Analysis of nuclear β-catenin accumulation before starting treatment might help predict the therapeutic response to NACRT.

## 1. Introduction

Neoadjuvant chemoradiotherapy (NACRT) followed by total mesorectal excision (TME) is the standard treatment for locally advanced rectal cancer. It has been reported that NACRT could improve locoregional control with local recurrence rates of approximately 5–9% [1,2,3]. However, response to NACRT varies widely among patients [4,5,6]. Some previous studies demonstrated that poor responders to NACRT had significantly worse oncological outcomes than good responders [7,8,9]. Moreover, poor responders may only have adverse effects with no benefits. Therefore, it is valuable to identify predictive biomarkers for a therapeutic response to NACRT.

Wnt/β-catenin signaling is known to play a crucial role in the regulation of cell proliferation, differentiation, and morphogenesis throughout the body [10,11,12]. Recently, inhibition of Wnt/β-catenin signaling has been found to significantly suppress the expression of colorectal cancer stem cell markers, such as CD44 and CD133, and tumorigenicity in immunodeficient mice [13]. This indicates that the activation of Wnt/β-catenin signaling is essential for the maintenance and proliferation of colorectal cancer stem cells, which are thought to have the ability to confer resistance to chemotherapy and radiotherapy by activating signaling pathways important for self-renewal [14,15,16]. Several investigators studied the possible role of the Wnt/β-catenin signal in chemoradiotherapy resistance or oncological outcomes in patients with rectal cancer undergoing NACRT followed by surgery [17,18,19]. However, its role remains unclear.

In the present study, the association of Wnt/β-catenin signal activation with a pathological response to NACRT in patients with locally advanced low rectal cancer was investigated. In addition, we investigated its association with long-term outcomes in such patients.

## 2. Materials and Methods

### 2.1. Study Population

Overall, 70 patients with locally advanced rectal cancer who received curative surgery following NACRT at Kobe University Hospital from January 2005 to July 2020 were retrospectively analyzed in this study. Inclusion criteria for this study were as follows: histologically proven adenocarcinoma, lower tumor margin below the peritoneal reflection, and cT3/4 or cN+ disease without distant metastasis. In contrast, patients whose biopsy specimens before NACRT were not available for immunohistochemical examination were excluded. Finally, 60 patients were subjected to the final analysis. Tumors were classified according to the tumor-node-metastasis system by the American Joint Committee on Cancer [20].

The Institutional Review Board and Ethics Committee of the Kobe University Graduate School of Medicine (IRB reference number: B210041) provided their approval for this study to be conducted.

### 2.2. Treatment Strategy

Patients with locally advanced low rectal cancer received NACRT comprising a total radiation dose of 45–50 Gy in 25 fractions for 5 weeks as well as an oral 5-fluorouracil-based chemotherapy (tegafur-uracil/leucovorin or capecitabine), as described previously [9,21]. 45 Gy radiotherapy with tegafur-uracil/leucovorin or 50 Gy radiotherapy with capecitabine was administered. Tegafur–uracil 200 mg/m^2^/day and leucovorin 75 mg/body/day or capecitabine 1650 mg/m^2^/day were orally administered for 25 days. Concomitant chemotherapy was initiated on the first day of radiotherapy. The lateral pelvic area was included in the radiation target volume. Surgery according to the TME principle was performed 6–8 weeks after the completion of NACRT. The lateral pelvic lymph node dissection was performed only in patients with clinically positive lateral pelvic lymph nodes based on the pretreatment images, regardless of the clinical response to NACRT. Clinically positive metastasis of the lateral pelvic lymph node was diagnosed by a short axis diameter of ≥7 mm on computed tomography or magnetic resonance imaging scans and/or a high-intensity spot on a positron emission tomography scan. Patients found to have developed distant metastases on post-NACRT imaging studies were excluded from the group of those indicated for curative surgery.

The pathological tumor response to NACRT was determined based on the grading scale according to the Japanese Society for Cancer of the Colon and Rectum guidelines [22]. Briefly, grades 0, 1a, 1b, 2, and 3 correspond to no response to treatment, 1/3 tumor size reduction, 1/3–2/3 tumor size reduction, >2/3 tumor size reduction, and complete tumor ablation, respectively. Notably, grade 3 corresponds to a pathological complete response. In the present study, patients with grades 0, 1a, and 1b were classified as poor responders, and those with grades 2 and 3 were classified as good responders.

Postoperatively, adjuvant chemotherapy was considered for all patients, regardless of the pathological stage, and follow-up was performed every 3 months for the first 3 years and every 6 months thereafter, as previously reported.

### 2.3. β-Catenin Immunostaining

Biopsy samples collected before NACRT were fixed in formalin and embedded in paraffin for immunohistochemistry. Serial cross-sections were cut and stained with hematoxylin and eosin to identify the most representative part of the tumor. β-catenin immunostaining was performed on formalin-fixed paraffin-embedded tissue according to the manufacturer’s instructions. Further, the β-catenin-specific monoclonal antibody (FLEX monoclonal mouse anti-human b-catenin, clone b-catenin-1; Dako, Santa Clara, CA, USA) was used to detect β-catenin.

**Figure 1:**
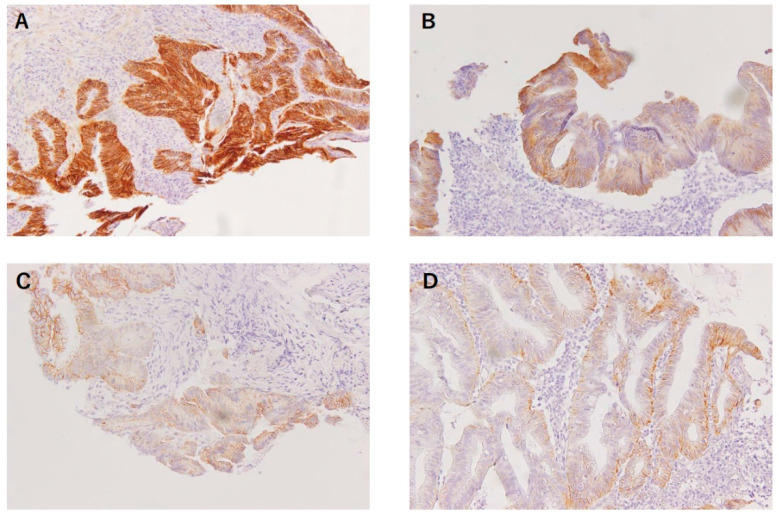
Nuclear β-catenin expression in biopsied rectal cancer samples before NACRT. Tumor cells corresponding to score 0 (<1% of positive cells) (**A**), score 1+ (1–5% of positive cells) (**B**), score 2+ (5–30% of positive cells) (**C**) and score 3+ (>30% of positive cells) (**D**).

The expression of β-catenin in the nucleus and membrane of the tumor cells was evaluated by a surgeon working in the pathological department and reviewed by a pathologist without knowledge of the clinical information. The expression of β-catenin in the nucleus or membrane was semi-quantitatively evaluated by calculating the ratio of the number of tumor cells that expressed nucleus or membranous β-catenin to the total number of tumor cells in the tissue section. Further, the ratio was scored as follows: 0 (<1% of positive cells), 1+ (1%–5% of positive cells), 2+ (5%–30% of positive cells), or 3+ (>30% of positive cells). According to the previous studies, the reference value of the ratio considered to be high nuclear β-catenin expression ranges from > 0 % to 30 % and has not been standardized [18,19,23,24]. In the present study, scores 0 and 1+ were classified as low nuclear β-catenin expression and scores 2+ and 3+ were classified as high nuclear β-catenin expression (Figure 1).

### 2.4. Statistical Analysis

The chi-square or Fisher’s exact test, as appropriate, was used to perform the comparison of categorical variables. Nonparametric variables were presented as median values and ranges. The Mann–Whitney U test was used to compare these variables. The Kaplan–Meier method was used to perform survival analysis, and the log-rank test was used to perform the univariate survival comparison. Univariate and multivariate analysis was performed to evaluate the predictive factor for relapse-free survival. Variables with a *p*-value < 0.1 in the univariate analysis were further subjected to the multivariate analysis. A *p*-value of <0.05 was considered statistically significant. All statistical analyses were performed with EZR 1.54 (Saitama Medical Center, Jichi Medical University, Saitama, Japan), which is a graphical user interface for R 4.2.2 (The R Foundation for Statistical Computing, Vienna, Austria).

## 3. Results

According to the nuclear β-catenin expression score, patients were classified into the low (*n* = 25) and high (*n* = 35) nuclear β-catenin expression groups. The low expression group included 3 and 22 patients with 0 and 1+ scores, respectively, whereas the high expression group included 18 and 17 patients with 2+ and 3+ scores, respectively. Patient and tumor characteristics are summarized in Table 1. No significant differences were observed between the groups in terms of each factor, except for the higher body mass index in the low expression group and higher rate of cT3 disease in the high expression group.

Operative outcomes are presented in Table 2. There were no significant differences in each factor between the groups. Furthermore, the postoperative outcomes, including the postoperative complications and recurrence rate, were similar between the groups (Table 3).

Regarding the pathological outcomes, patients in the high nuclear β-catenin expression group tended to have more advanced disease and a higher rate of positive vascular invasion than those in the low expression group (Table 4). Furthermore, the rate of good histological response was significantly higher in the low expression group (72% vs. 37.1%, *p* < 0.01).

**Figure 2:**
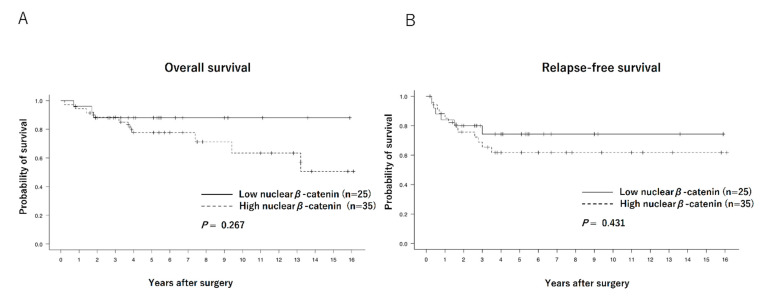
Overall survival curves (**A**) and relapse-free survival curves (**B**) of patients with low (*n* = 25) and high (*n* = 35) nuclear β-catenin expressions.

**Figure 3:**
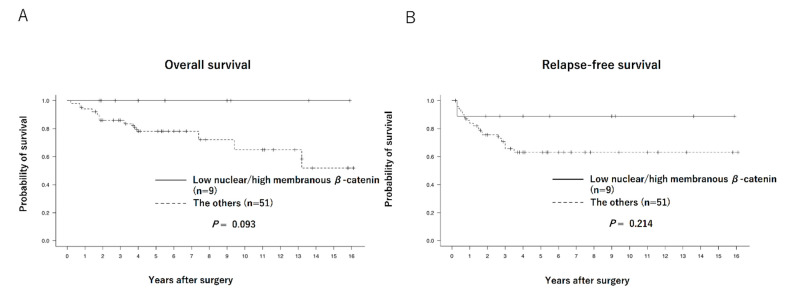
Overall survival curves (**A**) and relapse-free survival curves (**B**) of patients with low nuclear/high membranous β-catenin expression (*n* = 9) and others (*n* = 51).

The Kaplan–Meier curves of the overall survival (OS) and relapse-free survival (RFS) of each group are shown in Figure 2. No significant differences were observed between the low and high nuclear β-catenin expression groups in terms of OS (88.0% vs. 80.0%, *p* = 0.267) or RFS (76.0% vs. 62.8%, *p* = 0.431). Regarding comparison between patients with low nuclear β-catenin/high membranous β-catenin expression (*n* = 9) and others (*n* = 51), OS and RFS tended to be better in the former (100% vs. 76.4%, *p* = 0.093, and 88.9% vs. 64.7%, *p* = 0.214, respectively), although the differences were not statistically significant (Figure 3).

Table 5 shows univariate and multivariate analyses for RFS. No significant predictors were detected after multivariate analysis.

## 4. Discussion

NACRT followed by surgery remains a key treatment for patients with locally advanced rectal cancer, although recent advances in total neoadjuvant therapy appear promising [25,26,27,28]. However, their oncological outcomes depend on the response to NACRT, and there has been no reliable predictor available before treatment initiation. Activation of the Wnt/β-catenin signal pathway, represented by the accumulation of β-catenin in the nucleus, is essential for colorectal cancer stem cell maintenance and proliferation, suggesting that it contributes to resistance to NACRT [29,30,31,32]. In the present study, nuclear β-catenin expression was found to be associated with resistance to NACRT. Additionally, patients with nuclear β-catenin accumulation from the membrane had poorer oncological outcomes [33].

In the present study, high nuclear β-catenin expression was significantly associated with poor response to NACRT. However, its mechanism is still unclear. Takahashi et al. reported that nuclear β-catenin accumulation contributed to resistance to NACRT possibly through its regulation of cancer stem cells (CSC)/epithelial–mesenchymal transition (EMT) properties [19]. Therefore, we evaluated the expression of CD44, which was known to be one of the CSC markers. However, there was no association between the nuclear β-catenin and CD44 expression (data not shown). EMT, which plays a central role in converting epithelial cells into derivatives with a more mesenchymal phenotype, is also considered to contribute to a therapeutic response in colorectal cancer [34,35,36]. Several investigators have already reported that the Wnt/β-catenin signal regulated EMT in a wide variety of cancer cells [37,38,39]. Bhangu et al. reported that reduced expression of microRNA-200c—an upstream master-regulator of EMT—was significantly associated with nonresponse to NACRT [40]. Taken together, the Wnt/β-catenin signal might contribute to responses to NACRT through regulation of the EMT process in rectal cancer.

The present study could not demonstrate a significant association between the level of nuclear β-catenin expression and RFS or OS. However, importantly, better oncological outcomes were observed in the patients with low nuclear β-catenin/high membranous β-catenin levels. Recurrence developed only in one of those nine patients, and all of them were alive within a median follow-up period of 7 years. These results suggested that the localization and expression patterns of β-catenin have prognostic importance. β-catenin has been reported to be found in four distinct subcellular locations: the plasma membrane, cytoplasm, nucleus, and centrosomes [41]. Several studies have found that β-catenin localizes primarily to the plasma membrane in normal colon tissue; however, it exhibits decreased membranous and enhanced nuclear localization in colon cancers [24,42]. Nuclear β-catenin accumulation as well as its membrane dissociation and nuclear translocation may be important in maintaining resistance to NACRT in rectal cancer.

This study’s data imply the two possible roles of Wnt/β-catenin signal for patients with rectal cancer undergoing NACRT. First, evaluation of β-catenin expression using biopsy samples might enable the selection of good or poor responders before treatment initiation. Second, the utilization of the Wnt/β-catenin inhibitor might increase sensitivity to NACRT and improve the response rate. Recently, Leung et al. demonstrated that sulfasalazine—a niclosamide derivative anti-inflammatory drug—could suppress colorectal cancer stemness and metastasis by targeting Kirsten rat sarcoma virus signaling, which was involved in the activation of the Wnt/β-catenin pathway [43]. They proposed it as a possible adjuvant to improve chemotherapeutic responses in patients with colorectal cancer. Targeting the Wnt/β-catenin signal might be a promising approach to improve the sensitivity of rectal cancer to NACRT.

This study has several limitations. First, it is a retrospective small-scale single-institutional study. Second, the specimen was obtained from biopsy samples, and the invasive front of the tumor was not examined. It was hypothesized that EMT was mainly observed at the invasive front of the tumor [40], suggesting that Wnt/β-catenin activation should be observed at this site. Therefore, a biopsy sample might not be suitable for the evaluation of the association between the therapeutic response of the tumor and Wnt/β-catenin signal activation.

In conclusion, activation of the Wnt/β-catenin signal pathway represented by nuclear β-catenin accumulation was significantly associated with poor response to NACRT in patients with rectal cancer. Analysis of nuclear β-catenin accumulation before treatment initiation might help predict the therapeutic response to NACRT. Furthermore, the Wnt/β-catenin pathway was considered one possible therapeutic approach for improving the therapeutic response to NACRT.

## Figures and Tables

**Table 1:** Patient and tumor characteristics.

	*β*-Catenin Low	*β*-Catenin High	*p*
	*n* = 25	*n* = 35	
Age, mean (SD)	67.52 (9.91)	64.37 (8.30)	0.187
<65, *n* (%)	9 (36.0)	16 (45.7)	0.596
>65, *n* (%)	16 (64.0)	19 (54.3)	
<70	15 (60.0)	25 (71.4)	0.412
>70	10 (40.0)	10 (28.6)	
Sex, *n* (%)			
Male	19 (76.0)	24 (68.6)	0.575
Female	6 (24.0)	11 (31.4)	
BMI (kg/m^2^) ^†^	23.22 (17.8–30.6)	19.47 (15.8–30.7)	0.026
ASA-PS score, *n* (%)			0.936
1	12 (48.0)	18 (51.4)	
2	10 (40.0)	12 (34.3)	
3	3 (12.0)	5 (14.3)	
cT *, *n* (%)			
0, 1	1 (4.0)	0 (0.0)	0.004
2	4 (16.0)	0 (0.0)	
3	13 (52.0)	30 (85.7)	
4	7 (28.0)	5 (14.3)	
cN *, *n* (%)			
0	4 (16.0)	6 (17.1)	1.00
1	8 (32.0)	11 (31.4)	
2	13 (52.0)	18 (51.5)	
cM *, *n* (%)			
0	22 (88.0)	30 (85.7)	1.00
1	3 (12.0)	5 (14.3)	
cStage *, *n* (%)			
0–I	0 (0.0)	0 (0.0)	1.00
II	4 (16.0)	6 (17.1)	
III	18 (72.0)	24 (68.6)	
IV	3 (12.0)	5 (14.3)	
**Completion of NACRT**			
Yes	22 (88.0)	34 (97.1)	0.298
No	3 (12.0)	1 (2.9)	
**Adjuvant chemotherapy, *n* (%)**			
Yes	16 (64.0)	17 (36.4)	0.297
No	9 (36.0)	18 (63.6)	

ASA-PS, American Society of Anesthesiologists physical status; NACRT, neoadjuvant chemoradiotherapy. * Tumors were classified according to the American Joint Committee on Cancer (AJCC) TNM system. ^†^ The data are expressed as the median (range).

**Table 2:** Operative outcomes.

	*β*-Catenin Low	*β*-Catenin High	*p* Value
	*n* = 25	*n* = 35	
Operative procedure, *n* (%)			
HAR	0 (0.0)	1 (2.9)	0.958
LAR	7 (28.0)	10 (28.6)	
ISR	2 (8.0)	4 (11.4)	
APR	16 (64.0)	20 (57.1)	
Hartmann	0 (0.0)	0 (0.0)	
Surgical approach, *n* (%)			
Open	7 (28.0)	13 (37.1)	0.581
Laparoscopy	18 (72.0)	22 (62.9)	
**D, *n* (%)**			
D1	0 (0.0)	0 (0.0)	1.000
D2	2 (8.0)	3 (8.6)	
D3	23 (92.0)	32 (91.4)	
**LLND, *n* (%)**			
Yes	15 (60.0)	20 (57.1)	1.000
No	10 (40.0)	15 (42.9)	
**Operation time (min) ^†^**	489.24 (211–1052)	516.83 (244–1138)	0.609
**Estimated blood loss (ml) ^†^**	649.36 (0–4200)	603.91 (0–5345)	0.861
**Blood transfusion, *n* (%)**			
Yes	9 (36.0)	10 (28.6)	0.583
No	16 (64.0)	25 (71.4)	

HAR, high anterior resection; LAR, low anterior resection; ISR, intersphincteric resection; APR, abdominoperineal resection; LLND, lateral pelvic lymph node dissection. **^†^** The data are expressed as the median (range).

**Table 3:** Postoperative outcomes.

	*β*-Catenin Low	*β*-Catenin High	*p* Value
	*n* = 25	*n* = 35	
Postoperative complications (CD ≥ II), *n* (%)			
wound infection	2 (8.0)	3 (5.7)	1.000
wound dehiscence	0 (0.0)	0 (0.0)	NA
anastomotic leakage	1 (4.0)	4 (11.4)	0.390
bowel obstruction	1 (4.0)	2 (5.7)	1.000
lymphorrhea	2 (8.0)	1 (2.9)	0.565
deep vein thrombosis	0 (0.0)	0 (0.0)	NA
dysuria	2 (8.0)	4 (11.4)	1.000
ureteric injury	0 (0.0)	2 (5.7)	0.506
others	2 (8.0)	3 (8.6)	1.000
Postoperative complications (CD ≥ III), *n* (%)	7 (28.0)	10 (28.6)	1.000
**Postoperative hospital stay ^†^, days (range)**	41.48 (15–181)	50.20 (12–205)	0.406
**Mortality within 30 days, *n* (%)**	0 (0.0)	0 (0.0)	1.000
**Reoperation within 30 days, *n* (%)**	0 (0.0)	0 (0.0)	1.000
**Recurrence, *n* (%)**			
Yes	19 (76.0)	23 (65.7)	0.569
No	6 (24.0)	13 (34.3)	

CD, Clavien–Dindo classification. Sarcopenia was assessed by the total volume after NACRT. ^†^ The data are expressed as the median (range).

**Table 4:** Pathological outcomes.

	*β*-Catenin Low	*β*-Catenin High	*p* Value
	*n* = 25	*n* = 35	
ypT *, *n* (%)			
0-is	4 (16.0)	0 (0.0)	0.019
1	2 (8.0)	0 (0.0)	
2	6 (24.0)	8 (22.9)	
3	13 (52.0)	25 (71.4)	
4	0 (0.0)	2 (5.7)	
ypN *, *n* (%)			
0	17 (68.0)	19 (54.3)	0.441
1	5 (20.0)	8 (22.9)	
2	3 (12.0)	8 (5.7)	
ypM *, *n* (%)			
0	24 (96.0)	31 (88.6)	1.00
1	1 (4.0)	4 (11.4)	
ypStage *, *n* (%)			
0	4 (16.0)	0 (0.0)	0.0521
I	5 (20.0)	5 (14.3)	
II	8 (32.0)	14 (40.0)	
III	7 (28.0)	12 (34.3)	
IV	1 (4.0)	4 (8.75)	
Histological type *, *n* (%)			0.508
Well/moderately	21 (84.0)	32 (91.4)	
Mucinous/poorly	3 (12.0)	3 (8.6)	
Other	1 (4.0)	0 (0.0)	
Vascular invasion, *n* (%)			
Absent	20 (80.0)	15 (42.9)	0.007
Present	5 (20.0)	20 (57.1)	
Lymphatic invasion, *n* (%)			
Absent	20 (80.0)	25 (71.4)	0.552
Present	5 (20.0)	10 (28.6)	
Histological response ** (%)			
Poor (Grade 1a, 1b)	7 (28.0)	22 (62.9)	<0.01
Good (Grade 2, 3)	18 (72.0)	13 (37.1)	

* Tumors were classified according to the American Joint Committee on Cancer (AJCC) TNM system. ** According to the Japanese Society for Cancer of the Colon and Rectum guidelines.

**Table 5:** Univariate and multivariate analyses for relapse-free survival.

		Univariate Analysis		Multivariate Analysis	
Factor	*n*	HR	*p* Value	HR	*p* Value
Age (>70/<70)	20/40	0.598 (0.20–1.82)	0.364		
Sex (male/female)	42/18	2.637 (0.76–9.14)	0.126		
ASA-PS (>2/1,2)	8/52	1.075 (0.25–4.69)	0.923		
Postoperative Complication (CD ≥III/CD I, II)	17/43	0.881 (0.29–2.68)	0.824		
β-catenin staining, nuclear (High/Low)	35/25	1.444 (0.54–3.85)	0.462		
β-catenin staining, nuclear-cytoplasm (the others/Low-High)	51/9	3.324 (0.44–24.9)	0.243		
Pathological Response (Poor/Good)	29/31	2.629 (0.96–7.01)	0.054	2.322 (0.81–6.63)	0.115
Surgical Method (open/laparo)	20/40	2.168 (0.86–5.46)	0.101		
ypT (3,4/0,1,2)	40/20	1.372 (0.49–3.86)	0.548		
ypN (positive/negative)	24/36	3.276 (1.27–8.47)	0.014	2.524 (0.95–6.69)	0.062
Ly (present/absent)	15/45	2.354 (0.91–6.11)	0.078	1.941 (0.69–5.42)	0.205
V (present/absent)	25/35	2.804 (1.08–7.27)	0.034	1.440 (0.48–4.32)	0.515
Histology (por, muc/tub1, 2)	7/53	1.561 (0.45–5.40)	0.482		
Adjuvant chemotherapy (No/Yes)	27/33	1.313 (0.52–3.33)	0.566		

HR, hazard ration; ASA-PS, American Society of Anesthesiologists physical status; CD, Clavien–Dindo classification.

## Data Availability

The data presented in this study are available on request from the corresponding author.
